# Differences in health-related quality of life between three clusters of physical activity, sitting time, depression, anxiety, and stress

**DOI:** 10.1186/1471-2458-14-1088

**Published:** 2014-10-20

**Authors:** Amanda L Rebar, Mitch J Duncan, Camille Short, Corneel Vandelanotte

**Affiliations:** School of Human, Health, and Social Sciences, Central Queensland University, Building 18, Bruce Highway, Rockhampton, QLD 4702 Australia; School of Medicine & Public Health; Priority Research Centre for Physical Activity and Nutrition, Faculty of Health and Medicine, The University of Newcastle, University Drive, Callaghan, NSW 2308 Australia

**Keywords:** Mental health, Sedentary behaviour, Exercise, Cluster analysis

## Abstract

**Background:**

Physical inactivity, sitting behaviour, and mental health problems are detrimental to health-related quality of life but typically are considered as independent determinants. This study tested how these factors clustered together as profiles of subgroups of people and whether the clusters differed as a function of physical and mental health-related quality of life.

**Methods:**

In 2012, Australian adults (*N* =1,014) self-reported their physical and mental health-related quality of life, physical activity, sitting time, depression, anxiety, and stress using a web-based survey. Cluster analysis was used to identify subgroups of health behaviour and mental health profiles, and ANOVA was used to test for between-cluster differences in health-related quality of life.

**Results:**

Three subgroups were identified: people with higher psychological stress (*n* =13%), people with higher amounts of sitting time (*n* =45%), and people with lower amounts of sitting time (*n* =42%). There were no differences in mental health-related quality of life between subgroups; however people represented by the subgroup of higher amounts of sitting time had significantly lower physical health-related quality of life than the other two subgroups, *F*(2, 1011) =10.04, *p* < .01.

**Conclusions:**

Interventions should consider that (1) physical activity, sitting time, and psychological distress are aspects of multifaceted behavioural-psychological profiles, and (2) reductions of sitting time may have major impacts for physical health-related quality of life.

## Background

Psychological distress (i.e., depression, anxiety, stress symptoms), physical inactivity, and high amounts of sitting time are burdensome for health [1–3] and quality of life [4–7]. To this point, the research conducted on these topics has either focused on behaviour or psychological distress, but in reality, these constructs do not exist in isolation, but rather as an aspect of a person’s multifaceted behavioural-psychological profile [8]. The relevance and disseminability of this line of research can be improved with a more holistic perspective of how people’s physical activity, sitting behaviour and psychological distress (i.e., depression, anxiety, and stress) group together to impact health and quality of life. It may be that certain behavioural-psychological patterns characterize subgroups of people and some of these subgroups might have poorer health and quality of life than others.

A strong evidence-base shows that physical activity is associated with lower psychological distress [9–11], and emerging research is suggesting that sitting time is also associated with high psychological distress [12–14]. It is becoming increasingly clear that physical activity, sitting behaviour, and psychological distress are interrelated e.g., [10–15], but no previous research has examined how these factors cluster together to form profiles. There is a growing recognition that intervention strategies targeted at a well-defined group are more effective at promoting physical activity than ‘one-size-fits’ all approaches [16]. The identification of more holistic profiles can be used to inform the development and implementation of interventions.

Health-related quality of life (HRQoL) is useful as a global indicator of perceived health status and quality of life and it is strongly linked to risk of chronic disease [17, 18]. Not surprisingly, people with less psychological distress generally have better HRQoL than people with mental health symptoms [6, 7]. Additionally, people who are more physically active and spend less time sitting tend to have better HRQoL than their peers [3, 4, 19]. This previous research has focused on the isolated effects of these behavioural or psychological distress factors, but it remains unclear how these factors are linked to HRQoL when considered holistically as behavioural-psychological profiles. It may be, for example, that certain profiles are more strongly associated to HRQoL than others and, therefore, should be sought as a holistic goal of intervention efforts (rather than isolating one outcome to target).

The aims of the present study were twofold. The first aim was to identify population subgroups represented by behavioural (i.e., physical activity, sitting time) and psychological (i.e., depression, anxiety, and stress) profiles. It was hypothesized people who engaged in higher amounts of sitting time and lower amounts of physical activity would tend to have higher depression, anxiety, and stress. The second aim was to determine how the resultant subgroups of people differed in HRQoL. It was hypothesized that the subgroups characterized by higher amounts of sitting time, lower amounts of physical activity, and higher depression, anxiety, and stress would have poor HRQoL.

## Methods

### Participants and procedures

This study was conducted as part of the Australian Health and Social Science (AHSS) panel [20]. Australian adults were recruited with computer-assisted telephone interviewing and asked to participate in an online survey with questions about a range of socio-demographic and health-related topics. AHSS members (*N* = 3,932) were emailed a link to the online survey in August-September 2012. Study procedures were approved by the Central Queensland University Human Research Ethics Committee. Data were used from participants that responded to questions about their HRQoL, physical activity, sitting time, and mental health outcomes (*N* = 1,014).

### Measures

Physical activity was calculated as total minutes of walking, moderate physical activity, and vigorous physical activity (weighted by 2) of the previous week, as reported on the Active Australia Survey [21]. Participants were asked how many times they participated in these activities in bouts of ten minute or more and the typical time spent in these activities. Time spent in each of these activities was truncated to 14 hours per week and total activity time was truncated to 28 hours per week [21]. Previous research has demonstrated this measure has acceptable reliability and validity in adult men and women [22, 23].

Sitting time was calculated as the average time spent sitting daily in the past week, as reported on the Workforce Sitting Questionnaire [24]. On this 10-item measure, participants were asked how much time they spent sitting on work- and non-work days while working, commuting, using a computer, watching TV, and during other leisure-time activities. This measure has demonstrated acceptable reliability and validity [24].

Depression, anxiety, and stress were assessed with the Depression, Anxiety, and Stress Scale [25]. On this 21-item measure, participants reported how much the statements applied to them over the past week on a response scale from 0 (*did not apply to me at all*) to 3 (*applied to me very much, or most of the time*). Sample items include, “I felt that I had nothing to look forward to (*depression*),” “I felt scared without any good reason (*anxiety*),” and “I found it hard to wind down (*stress*).” The three 7-item scales had acceptable internal validity in the current study (depression α = .91, anxiety α = .77, stress α = .88) and have demonstrated satisfactory psychometric properties previously [25, 26].

HRQoL was assessed with the physical and mental component scores of the Veterans RAND 12-item Health Survey [27]. Participants reported how much physical/emotional health resulted in problems with work or regular daily activities. This scale was developed from the Veterans RAND 36-item Health Survey which was developed and modified from the original RAND version of the 36-item Health Survey version 1.0. Raw scores were transformed to standardized 0 – 100 scores (*M* = 50 and a *SD* = 10), in which 50 represents population norms [5]. Previous research has shown that the scales have satisfactory psychometric properties [27, 28].

### Data analysis

A two-step cluster analysis [29, 30] of Z-scores of physical activity, sitting time, depression, anxiety, and stress was conducted in *R* version 2.15 [31] to partition participants into subgroups of behavioural-psychological profiles. Cluster analysis creates groups in which the values of a set of variables for individuals in the same group are more similar to each other than to those of individuals in the other groups [32]. The similarity method used was the Euclidean distance. The resultant number of clusters was selected based on the distances of the hierarchical cluster dendogram, comparisons of internal validity and stability of alternative cluster solutions, and conceptual considerations [33]. The internal validation measures were the Dunn index [34] and the silhouette width [35]. The stability measures were the average proportion of non-overlap (APN) and the average distance (AD) [36, 37]. To test if clusters differed in their physical and mental HRQoL, a series of Analysis of Variance (ANOVA) and post-hoc Tukey Honestly Significant Differences Tests were conducted. Significance was set at *p* < .05 and Bonferroni corrections were made to account for multiple test comparisons.

## Results

On average, participants (58% female, *M ± SD* age = 51 ± 11, BMI = 27.33 ± 5.20) had low depression (2.97 ± 3.78), anxiety (1.76 ± 2.51), and stress (3.94 ± 3.69). They engaged in 366.68 ± 383.79 minutes (52.37 ± 54.86 min/day) of physical activity and spent 586 ± 211.87 minutes (9.77 ± 3.53 hours/day) sitting daily in the past week.

**Figure 1:**
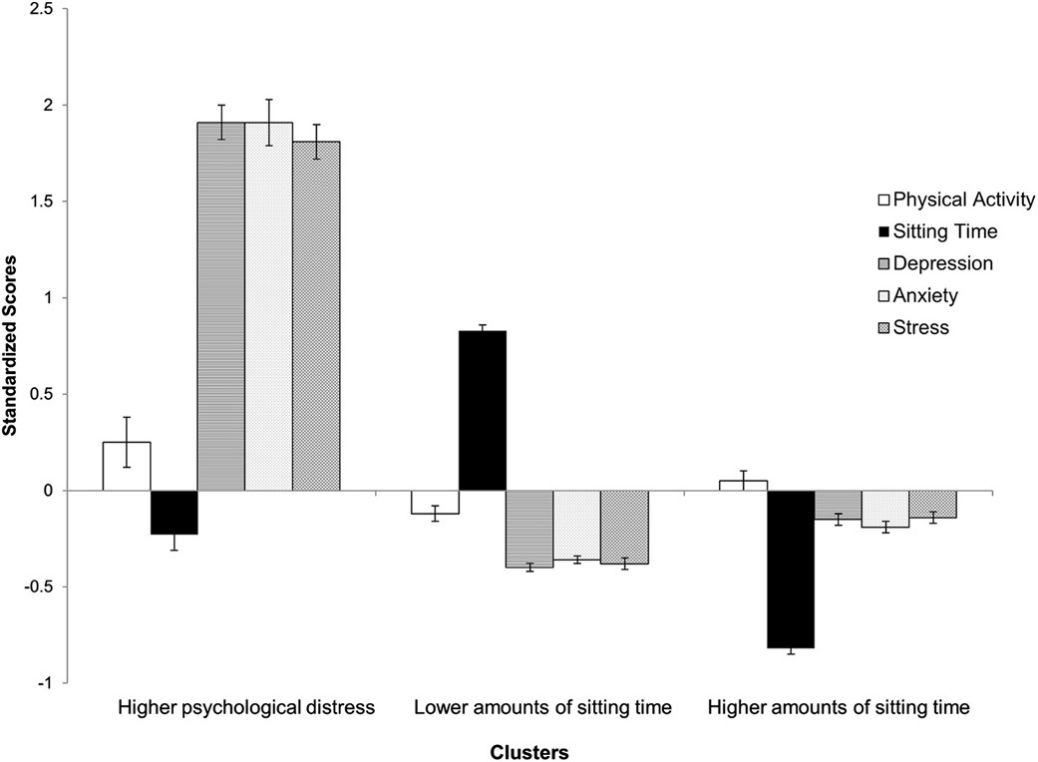
**Standardized (Z-)scores and standard error bars of physical activity, sitting time, depression, anxiety, and stress of the three clusters of data from Australian adults collected in 2012: (1) higher psychological distress, (2) higher amounts of sitting time, and (3) lower amounts of sitting time.**

Participants’ behavioural-psychological profiles could be partitioned into 3 subgroups. The resultant 3-cluster solution had better internal validity than the alternative 2-cluster or 4-cluster solutions (3-cluster: *Dunn*: 1.61, *Silhouette*: 0.25; 2-cluster: *Dunn*: 0.92, *Silhouette*: 0.25; 4-cluster: *Dunn*: 1.04, *Silhouette*: 0.26). The 3-cluster solution had better stability than the 2-cluster solution, but worse stability than the 4-cluster solution (3-cluster: *APN* = 0.00, *AD* = 11.01; 2-cluster: *APN* = 0.00, *AD* = 18.60; 4-cluster: *APN* = 0, *AD* = 4.87). The 3-cluster solution was retained because it had stronger internal validity and made more sense conceptually than the 4-cluster solution. Figure 1 presents the profiles of the three resultant clusters.

The first cluster (*higher psychological stress*) represented a small portion of the sample (*n* = 129, 13%) and was representative of people with moderate amounts of (i.e., near sample average) physical activity (*M*_*z*_ = 0.25, 66 min/day) and sitting time (*M*_*z*_ = -0.23, 8.96 hr/day), but higher (i.e., above average) depression (*M*_*z*_ = 1.91), anxiety (*M*_*z*_ = 1.91), and stress (*M*_*z*_ = 1.81). The second cluster (*higher amounts of sitting time*) represented the largest portion of the sample (*n* = 456, 45%). It was representative of people with moderate physical activity (*M*_*z*_ = -0.12 = 46.29 min/day), higher sitting time (*M*_*z*_ = 0.83 = 12.71 hr/day), and moderate depression (*M*_*z*_ = -0.40), anxiety (*M*_*z*_ = -0.36), and stress (*M*_*z*_ = -0.38). The third cluster (*lower amounts of sitting time*) represented the second largest portion of the sample (*n* = 429, 42%), and it was representative of people with moderate physical activity levels (*M*_*z*_ = 0.05 =  46.29 min/day), lower amounts of sitting time (*M*_*z*_ = -0.82 = 6.88 hr/day), and moderate depression (*M*_*z*_ = -0.15), anxiety (*M*_*z*_ = -0.19), and stress (*M*_*z*_ = -0.14).

**Figure 2:**
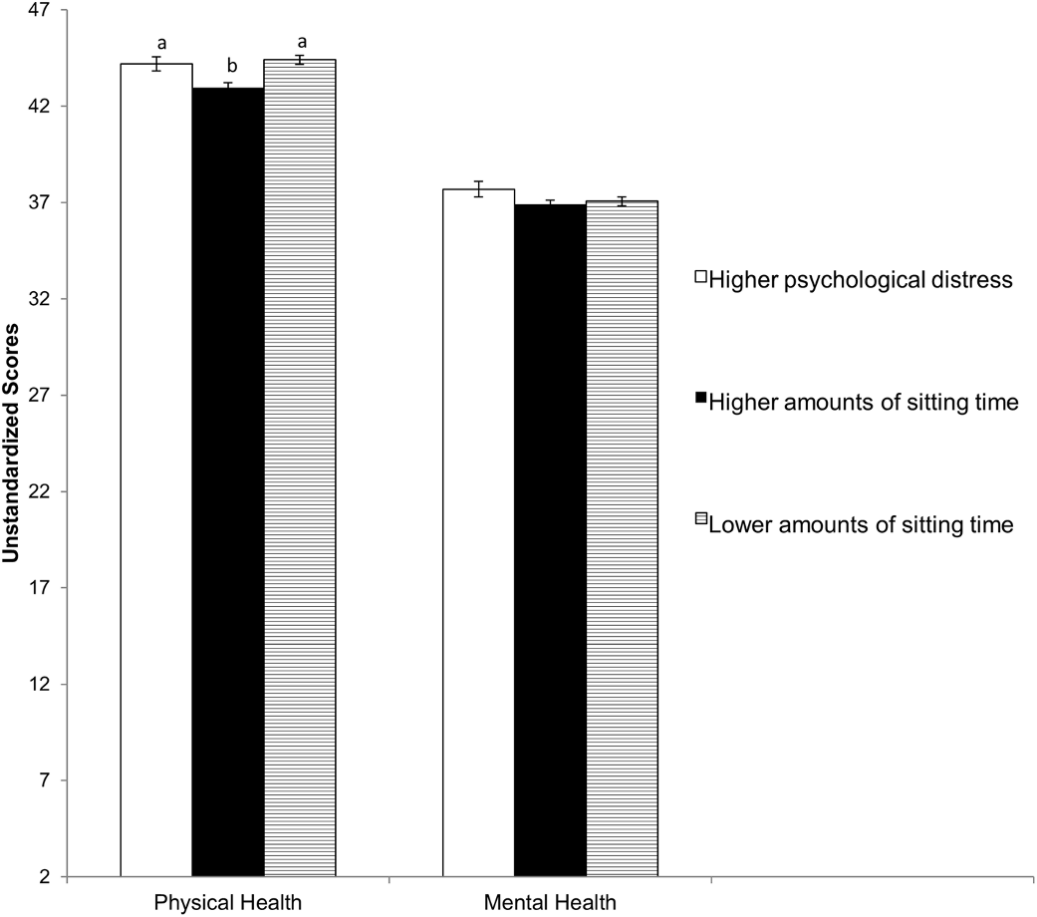
**Physical and mental health-related quality of life of each of the three clusters of data from Australian adults collected in 2012: (1) higher psychological distress, (2) higher amounts of sitting time, and (3) lower amounts of sitting time.** Physical health-related quality of life was significantly lower for the higher amounts of sitting time cluster (labelled with b) than for the other two clusters (labelled with a).

Figure 2 presents physical and mental HRQoL as a function of the three clusters. The clusters did not differ in mental HRQoL, *F*(2, 1011) = 1.35, *p* > .05, but were found to significantly differ in physical HRQoL, *F*(2, 1011) = 10.04, *p* = .03. The *higher amounts of sitting time* cluster had significantly lower physical HRQoL than the *higher psychological stress* cluster (95% CI of difference *= -*2.39 – -0.07) and the *lower amounts of sitting time* cluster (95% CI of difference *= -*1.44 – -0.66). Based on the population norms, these group differences have effect sizes of *d* = .12 and *d* = .04, respectively [28].

## Discussion

The aims of this paper were to (1) identify subgroups of people represented by behavioural-psychological profiles, and (2) test whether people in these subgroups differed in their physical or mental HRQoL. Three subgroups were identified: people with higher psychological distress; people with higher amounts of sitting time; and people with lower amounts of sitting time. There were no differences in mental HRQoL between subgroups; however people represented by the subgroup of higher amounts of sitting time had significantly lower physical HRQoL than the other two subgroups.

The profile represented by people with higher psychological distress corresponds with previous evidence suggesting high co-presence of these mental health problems [26]. This subgroup was representative of only a small portion (13%) of the sample, aligning with Australian prevalence rates of anxiety (14%) and depression (6.2%) [38]. Most people were represented by the other two subgroups of either higher or lower amounts of sitting time. Physical activity levels were moderate for both of these subgroups, supporting previous research that showed that sedentary behaviour and physical activity are not necessarily oppositional behaviours and may impact health outcomes in different ways [39, 40].

The subgroup representative of higher psychological distress was not profiled as physically inactive, which seems contrary to the typically low rates of physical activity typically found in populations with mental health problems [9–11]. The results of the present study, along with the strong evidence-base of the intervention effects of physical activity on depression, anxiety, and stress [11, 41, 42] lead to the conclusion that the commonly found negative association between physical activity and mental health problems is likely a consequence of a within-person change across time, such as a treatment effect (i.e., more physical activity results in less mental health problems), rather than a stable profile (i.e., people either have more psychological distress and are not physically active or have less psychological distress and are physically active). Higher amounts of sitting time did not group with psychological distress in the present study which may suggest that the connection between sitting time and mental health is also not the result of a stable profile. More research, however, is necessary to test the causality of this link.

The subgroups did not differ in their mental HRQoL, but the subgroup with higher amounts of sitting time had poorer physical HRQoL than the other subgroups. Contrary to previous research showing that mental health problems are detrimental to quality of life and wellbeing [6, 7], the subgroup with higher psychological distress did not significantly differ in mental HRQoL from the other two subgroups in the present study. The results of this study highlight that mental HRQoL is conceptually distinct from psychological distress. Mental HRQoL reflects how emotional and mental health problems interfere with functional capacity and wellbeing [18]. It may be that within-person changes in mental HRQoL and psychological distress are linked, but it seems that mental HRQoL does not vary between subgroups of varying behavioural-psychological profiles. Alternatively, it may be that the measures used in this study to assess depression, anxiety, and stress or HRQoL were not sensitive enough to reflect the true overlapping variability in psychological distress and mental HRQoL. Future research utilizing different measures are needed to rule out this alternative explanation.

High levels of uninterrupted sedentary behaviour increases the odds of poor cardiovascular and metabolic health and increase the risk of early mortality when adjusting for physical activity [40, 43–45]. The subgroup with higher amounts of sitting time had poorer physical HRQoL than the other subgroups, supporting this growing body of evidence linking sitting time with detrimental health. Prospective studies further investigating the relations between these constructs will help untangle the time ordering of these effects, and interventions targeting physical health status and quality of life may benefit from promoting reductions of people’s sitting time.

## Conclusions

The findings of this study generalize to a community Australian sample with fairly low psychological distress, high amounts of physical activity, and modest amounts of sitting time. Study participants were voluntarily involved in a web-based survey, so it may be that the sample was highly motivated; however no information was available on non-respondents so it was not possible to test for differences between those who did and did not respond to the survey. Depression, anxiety, and stress [25, 26] and amounts of sitting time [24] were similar to those of previous research of community samples, but the present study participants tended to participate in more physical activity than is representative of the general population [23], so it is also important to test the replicability of these results in a less active population.

Additionally, physical activity was assessed with a self-report measure that did not account for occupational physical activity, so future research is needed to determine whether the findings differ when objective assessment of behaviour or self-report measures more inclusive of multiple domains of physical activity are utilised. Most importantly, future research is necessary to determine how these results can be implemented to improve the way mental health and physical activity interventions target and treat people. People may respond to interventions differently as a function of these behavioural-psychological profiles. Future research could identify these profiles in prospective intervention participants and track intervention progress as a function of these subgroups.

Overall, the results of this study add to the literature that: 1 – high depression, anxiety, and stress tend to group together as a profile, suggesting that interventions targeting psychological distress need to consider targeting these mental health factors holistically, rather than in isolation; 2 – there were not significant differences in physical activity between people with higher vs. lower amounts of sitting time, suggesting that interventions targeting physical activity and/or sitting behaviour need to consider these behaviours as distinct, rather than oppositional, behaviours; and 3 – sitting time is linked with adverse physical HRQoL, even beyond that of higher psychological distress, suggesting that limiting sitting time could be important in health-focused intervention efforts.
